# Bisphenol A Exposure during Pregnancy Disrupts Glucose Homeostasis in Mothers and Adult Male Offspring

**DOI:** 10.1289/ehp.1001993

**Published:** 2010-05-19

**Authors:** Paloma Alonso-Magdalena, Elaine Vieira, Sergi Soriano, Lorena Menes, Deborah Burks, Ivan Quesada, Angel Nadal

**Affiliations:** 1 Centro de Investigación Biomédica en Red de Diabetes y Enfermedades Metabólicas Asociadas (CIBERDEM) and; 2 Instituto de Bioingeniería, Universidad Miguel Hernández de Elche, Elche, Spain; 3 Instituto Principe Felipe, Consejo Superior de Investigaciones Científicas, Valencia, Spain

**Keywords:** bisphenol A, diabetes, endocrine disruptors, estrogen, gestational diabetes, islet of Langerhans, pregnancy, xenoestrogens

## Abstract

**Background:**

Bisphenol A (BPA) is a widespread endocrine-disrupting chemical used as the base compound in the manufacture of polycarbonate plastics. In humans, epidemiological evidence has associated BPA exposure in adults with higher risk of type 2 diabetes and heart disease.

**Objective:**

We examined the action of environmentally relevant doses of BPA on glucose metabolism in mice during pregnancy and the impact of BPA exposure on these females later in life. We also investigated the consequences of *in utero* exposure to BPA on metabolic parameters and pancreatic function in offspring.

**Methods:**

Pregnant mice were treated with either vehicle or BPA (10 or 100 μg/kg/day) during days 9–16 of gestation. Glucose metabolism experiments were performed on pregnant mice and their offspring.

**Results:**

BPA exposure aggravated the insulin resistance produced during pregnancy and was associated with decreased glucose tolerance and increased plasma insulin, triglyceride, and leptin concentrations relative to controls. Insulin-stimulated Akt phosphorylation was reduced in skeletal muscle and liver of BPA-treated pregnant mice relative to controls. BPA exposure during gestation had long-term consequences for mothers: 4 months postpartum, treated females weighed more than untreated females and had higher plasma insulin, leptin, triglyceride, and glycerol levels and greater insulin resistance. At 6 months of age, male offspring exposed *in utero* had reduced glucose tolerance, increased insulin resistance, and altered blood parameters compared with offspring of untreated mothers. The islets of Langerhans from male offspring presented altered Ca^2+^ signaling and insulin secretion. BrdU (bromodeoxyuridine) incorporation into insulin-producing cells was reduced in the male progeny, yet β-cell mass was unchanged.

**Conclusions:**

Our findings suggest that BPA may contribute to metabolic disorders relevant to glucose homeostasis and that BPA may be a risk factor for diabetes.

During the last decade research has revealed that conditions experienced during early development play an important role in determining the long-term health of individuals. Alterations in development due to impaired maternal metabolism can lead to the permanent programming of physiological systems. Gestation generates a state of increased metabolic demand to ensure a balance between maternal and fetal requirements. To meet the demands of pregnancy, a coordinated series of maternal adaptations occur, including changes of metabolic processing within different tissues and changes in nutrient partitioning that ensure proper growth of the fetus (Ryan 2003). The most profound of these adaptations occurs with glucose metabolism because glucose is the primary nutrient for fetal growth and milk synthesis. Thus, glucose production increases during late pregnancy and early lactation, and concurrently, glucose uptake by muscle and adipose tissue progressively declines. This insulin-resistant state ensures that an adequate supply of glucose is shunted to the growing fetus (Barbour et al. 2007; King 2006). Despite this condition, serum glucose concentrations are maintained within the physiological range because the maternal endocrine pancreas adapts by increasing insulin secretion. If this adjustment fails, gestational diabetes ensues (Kuhl 1998). Experimental and epidemiological data suggest that gestational diabetes may have long-term consequences for both baby and mother, including a predisposition to obesity, metabolic syndrome, and diabetes later in life (Boloker et al. 2002; Reece et al. 2009).

In addition to insulin, other hormones change significantly in response to gestation. The rise of maternal serum levels of prolactin, placental lactogens, progesterone, and estradiol (E2) in late pregnancy is related, at least in part, to the development of insulin resistance (Gonzalez et al. 2003; Nadal et al. 2009b). Among these hormonal adaptations, those related to E2 are key. In addition to its role in the physiology of reproduction, E2 has been proposed to mediate maternal adaptation to the enhanced demand for insulin because it enhances insulin biosynthesis as well as glucose-stimulated insulin secretion (Nadal et al. 2009b). Although physiological levels of E2 are involved in maintaining normal insulin sensitivity (Liu and Mauvais-Jarvis 2010; Louet et al. 2004), E2 outside of the physiological range may have adverse effects on glucose homeostasis (Livingstone and Collison 2002; Nadal et al. 2009a).

Recently, environmental estrogens such as bisphenol A (BPA) have become public health concerns because of experimental evidence indicating deleterious effects on energy balance and glucose homeostasis in animal models (Alonso-Magdalena et al. 2006, 2008; Nadal et al. 2009a; Newbold et al. 2008). In humans, BPA has been associated epidemiologically with type 2 diabetes and heart disease (Lang et al. 2008). Although first discovered as a synthetic estrogen (Dodds and Lawson 1936), BPA is currently used as the base compound in the manufacture of polycarbonate plastic and the resin lining of food and beverage cans and drinking water bottles and containers (vom Saal et al. 2007). Importantly, BPA has been shown to leach from polycarbonate containers, and consequently, BPA has been widely detected in humans. Indeed, the potential risk for BPA exposure is emphasized by the finding of Calafat et al. (2008) that BPA was present in 92.6% of the urine samples from U.S. residents. The concentration of BPA in human serum ranges from 0.2 to 1.6 ng/mL (0.88–7.0 nM) (Vandenberg et al. 2007). In addition, it is has been detected in amniotic fluid, neonatal blood, placenta, cord blood, and human breast milk, demonstrating the potential of this compound to pass from mother to fetus (Vandenberg et al. 2007). Many *in vivo* and *in vitro* studies have reported adverse effects associated with BPA, and interestingly, many were caused by concentrations below the predicted “safe” reference dose of 50 μg/kg/day established by the U.S. Environmental Protection Agency (EPA) (vom Saal et al. 2007). In the present study, we chose low doses of BPA based on the U.S. EPA criterion for low-dose effects of endocrine- disrupting chemicals (EDCs). Levels below the current lowest observed effect level (LOAEL) of 50 mg/kg/day have been considered low dose for *in vivo* studies (Wetherill et al. 2007; U.S. EPA 2010). Although the initial concerns about BPA were related to reproductive parameters and its carcinogenic potential, few studies have examined the consequences of BPA exposure during pregnancy on the mother, and no study has assessed the potential risk for developing diabetes, despite the fact that gestational diabetes is a major potential complication of pregnancy with adverse consequences for both mothers and newborns.

In the present study, we demonstrate that low concentrations of BPA have deleterious long-term effects on glucose metabolism in mice during pregnancy and postpartum, as well as in their adult offspring. Our results suggest that BPA exposure could contribute to the development of gestational diabetes, obesity, and a prediabetic state later in life. Notably, *in utero* exposure to BPA was associated with decreased glucose tolerance and increased insulin resistance in male offspring at 6 months of age compared with controls, consistent with an effect of BPA on fetal programming that could predispose adult mice to type 2 diabetes and metabolic disorders.

## Materials and Methods

### Animals and treatment

Pregnant OF-1 mice (F_0_) were purchased from Charles River (Barcelona, Spain) and individually housed under standard housing conditions. Mice were maintained on 2014 Teklad Global 14% Protein Rodent Maintenance Diet (Harlan Laboratories, Barcelona, Spain), which does not contain alfalfa or soybean meal. The composition of the diet is as follows: crude protein, 14.3%; fat, 4%; carbohydrate, 48%; crude fiber, 4.1%; neutral detergent fiber, 18%; ash, 4.7%; energy density, 2.9 kcal/g (12.1 kJ/g); calories from protein, 20%; calories from fat, 13%; and calories from carbohydrate, 67%.

Experimental procedures were reviewed and approved by the institutional committee for animal care and use of the Universidad Miguel Hernández de Elche. Animals were treated humanely and with regard for alleviation of suffering.

BPA was dissolved in tocopherol-stripped corn oil and administered subcutaneously on days 9–16 of gestation (GD9–GD16). The daily dose used was 10 or 100 μg/kg. We observed no significant difference in litter size between control and BPA-treated mice. Pups of the same treatment group were pooled together and then placed in equal numbers (11 pups/group) with foster mothers of the same treatment group; pups housed together were of the same sex. Pups were weighed on postnatal days 1, 3, 5, 8, 10, 12, 16, and 21. Mean body weight was calculated as the mean of individual body weight of each pup per group per day. Animals were weaned on postnatal day 21 and housed (8 mice/group) from weaning through adulthood.

### Glucose and insulin tolerance tests

For the intraperitoneal glucose tolerance tests (IPGTT) animals were fasted overnight for 12 hr, and blood samples were obtained from the tail vein. Animals were then injected intraperitoneally with glucose at 2 g/kg body weight, and blood samples were taken at the indicated intervals (0, 15, 30, 60, and 120 min). For the intraperitoneal insulin tolerance tests (IPITT), fed animals were injected intraperitoneally with soluble insulin at 0.75 or 1.25 IU/kg body weight. Blood glucose was measured in each sample after 0, 15, 30, 45, and 60 min using an Accu-Chek compact glucometer (Roche, Madrid, Spain).

### Plasma analysis

To measure plasma metabolites, mice were anesthetized with sodium pentobarbital at 50 mg/kg body weight. Blood was obtained by cardiac puncture. Insulin and leptin were measured by enzyme-linked immunosorbent assay (Mercodia, Uppsala, Sweden, and Crystal Chem, Downers Grove, IL, USA, respectively), and triglycerides and glycerol were measured with the GTO-Trinder Triglycerides assay (Sigma, Madrid, Spain).

### Insulin secretion measurement

Pancreatic islets of Langerhans from 6-month-old F_1_ mice were isolated by collagenase (Sigma) digestion as previously described (Alonso-Magdalena et al. 2006). Islets were collected with a micropipette one by one and were used immediately after isolation. Islets were washed twice with a buffer solution containing 120 mM NaCl, 25 mM NaHCO_3_, 5 mM KCl, 2.5 mM CaCl_2_, 1 mM MgCl_2_, and 3 mM d-glucose (final pH of 7.35). Groups of five islets were then incubated in 1 mL of this buffer in the presence of 3, 7, or 16 mM glucose. After 1 hr, the medium was collected, and insulin was measured in duplicate samples by radioimmunoassay using a Coat-a-Count kit (Diagnostic Products Corp., Los Angeles, CA, USA). Protein concentration was measured by the Bradford dye method (Bradford 1976).

### Recording [Ca^2+^]_i_ (intracellular calcium concentrations)

Isolated islets of Langerhans were loaded with 5 μM fura 2-acetoxymethyl ester for at least 1 hr at room temperature. We obtained calcium records for the whole islet of Langerhans by imaging intracellular calcium under an inverted epifluorescence microscope (Axiovert 200; Carl Zeiss GmbH, Jena, Germany). Images were acquired every 2 sec with an extended Hamamatsu Digital Camera (model C4742-95; Hamamatsu Photonics, Barcelona, Spain) using a dual-filter wheel equipped with 340 and 380 nm, 10-nm bandpass filters. Data were acquired using AquaCosmos software from Hamamatsu. Fluorescence changes are expressed as the ratio of fluorescence at 340 and 380 nm (F_340_/F_380_). Results were plotted and analyzed with use of commercially available software (SigmaPlot, version 8.0; SPSS Inc., Chicago, IL, USA).

### Western blots

We conducted insulin signaling experiments for Western blot analysis. Briefly, pregnant mice were fasted 4 hr and administered a single intraperitoneal injection of insulin (0.6 U/kg); tissues were harvested 10 min later. Gastrocnemius muscles and liver were homogenized in ice-cold buffer [10% glycerol, 20 mM sodium pyrophosphate, 150 mM NaCl, 50 mM HEPES (pH 7.5), 1% NP-40, 20 mM β-glycerophosphate, 10 mM sodium fluoride, 1 mM EDTA, 1 mM EGTA, 2 mM phenylmethylsulfonyl fluoride, 10 μg/mL aprotinin, 10 μg/mL leupeptin, 2 mM sodium orthovanadate, 3 mM benzamidine (pH 7.4)] for 20 sec. Homogenates were rotated end over end for 1 hr at 4°C and subjected to centrifugation (14,000 × *g* for 10 min) at 4°C. Protein content in lysates was measured by the Bradford method (Bradford 1976). Muscle and liver lysates were adjusted to equal protein concentration, boiled in Laemmli buffer, and loaded on 7.5% gels. Membranes were blocked in Tris-buffered saline/Tween (TBST) buffer (10 mM Tris-base, 150 mM NaCl, 0.25% Tween 20) containing 5% low-fat milk protein for 2 hr at room temperature. Membranes were then incubated with primary antibodies overnight at 4°C, washed with TBST buffer, and incubated with appropriate horseradish peroxidase-conjugated secondary antibody (Bio-Rad, Richmond, CA, USA) for 1 hr at room temperature.

We determined Akt phosphorylation by using an antibody against phospho-Akt [threonine^308^ (Thr^308^); 1:1,000; Cell Signaling, Danvers, MA, USA]. An anti-Akt antibody was used to confirm equal loading and normalize samples. Protein bands were revealed using the Pierce ECL chemiluminescence kit (Amersham Biosciences, Barcelona, Spain). Intensity of the bands was quantified using Scion Image software (Scion, Frederick, MD, USA).

### Assessment of pancreatic β-cell area

Pancreata were removed from F_1_ mice at the time of sacrifice (6 months of age) and fixed overnight in 4% paraformaldehyde. Subsequently, pancreatic tissue was embedded in paraffin, and 5-μm sections were prepared. After rehydration and permeabilization (1% Triton X-100), sections were incubated with anti-insulin and anti-glucagon antibodies (both from Sigma) overnight at 4°C. Detection was performed with rhodamine- and fluorescein-conjugated secondary antibodies (Jackson Immunoresearch, Suffolk, UK). For quantification of β-cell area, sections were viewed at a magnification of 10×. We measured the cross-sectional area of the islet and the total pancreatic area sing the ImageJ analysis program (National Institutes of Health, Bethesda, MD, USA). At least three sections, separated by 200 μm, were measured per animal. For quantification of the number of islets per area, only islets with more than five positive-stained cells were scored.

### Analysis of bromodeoxyuridine (BrdU) incorporation

Mice (the same mice also used for assessment of pancreatic β-cell area) were given intraperitoneal injections of BrdU (100 μg/g) 4 hr before sacrifice. Pancreatic tissue was collected, fixed, and processed as described above. After dehydration, sections were heated to 100°C in the presence of citrate buffer (10 mM) for 5 min. Slides were then blocked by incubating for 30 min in 0.1% bovine serum albumin and 5% normal goat serum in phosphate-buffered saline/0.2% TX-100. Samples were then incubated with antibodies for insulin (1:200, rabbit polyclonal; Santa Cruz Biotechnology, Madrid, Spain) and BrdU (1:200, monoclonal; DAKO, Barcelona, Spain) overnight at 4°C. After incubation with secondary antibodies, sections were mounted using Fluorsave (Calbiochem, Madrid, Spain). Images were acquired from double-stained sections. BrdU-positive nuclei were scored only in cells that were also positive for insulin. Quantification was done on at least three sections, separated by 200 μm, from each animal.

### Statistical analysis

**Figure 1:**
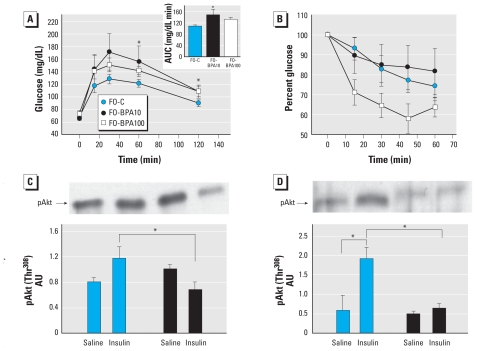
BPA exposure during pregnancy on blood glucose homeostasis in F_0_ mice. (*A*) IPGTT performed in F0-C (*n* = 11), F0-BPA10 (*n* = 8), and F0-BPA100 (*n* = 8); the inset shows mean total AUC in response to glucose load. (*B*) IPITT in F0-C (*n* = 12), F0-BPA10 (*n* = 9), and F0-BPA100 (*n* = 7) mice. (*C* and *D*) Western blots (top) and analysis (bottom) of insulin-stimulated Akt phosphorylation (Thr^308^; pAkt) in liver (*C*) and in gastrocnemius muscle (*D*); tissue for these experiments was collected 15 min after intraperitoneal injection of insulin (0.6 U/kg) or saline in F0-C (*n* = 4) and F0-BPA10 (*n* = 6) mice. Values for analysis are in arbitrary units (AU). Data are expressed as mean ± SE. *p < 0.05.

SigmaStat 3.1 software (Systat Software, Inc., Chicago, IL, USA) was used for all statistical analyses. To assess differences between treatment groups for each exposure paradigm, we used one-way analysis of variance (ANOVA) followed by the Bonferroni post hoc test. Results in Figure 1C and D were analyzed by two-way ANOVA followed by the Fisher least significant difference test. When data did not pass the parametric test, we used ANOVA on ranks followed by Dunn’s test (noted in figure legends). Results were considered significant at *p* < 0.05. All results are expressed as mean ± SE

## Results

### BPA exposure during pregnancy: consequences for maternal glucose homeostasis

To examine the effects of BPA on maternal glucose metabolism, we treated pregnant mice with either vehicle [controls, F0-C] or BPA at doses of 10 μg/kg/day (F0-BPA10) or 100 μg/kg/day (F0-BPA100) on GD9–GD16. We then measured glucose and insulin sensitivity and plasma metabolites on GD16–GD18. Across the groups, we matched animals for gestation day to minimize potential differences in the background levels of maternal hormones during the last phase of pregnancy.

Results of the IPGTT revealed that F0-BPA10 mice displayed glucose intolerance compared with F0-C mice (Figure 1A). The F0-BPA100 group displayed a tendency to glucose intolerance, yet the area under the curve (AUC), an index of glucose tolerance, was not significantly different than for vehicle-treated animals (Figure 1A, inset). We performed IPITTs to assess insulin sensitivity. In both F0-C and F0-BPA10 mice, insulin caused only a modest decrease in serum glucose levels, reflecting the physiological insulin resistance that develops during late pregnancy. However, we observed a tendency to increased insulin sensitivity in F0-BPA100 mice, although this did not reach statistical significance (Figure 1B).

Because BPA treatment during pregnancy led to altered glucose homeostasis, particularly in the F0-BPA10 group, we next studied signaling pathways in liver and skeletal muscle of pregnant mice that had received intraperitoneal injections of insulin. In liver from F0-C mice, insulin increased the phosphorylation of Akt compared with saline treatment (Figure 1C). In contrast, insulin stimulation actually decreased Akt phosphorylation in liver of F0-BPA10 mice, suggesting that BPA treatment impairs hepatic insulin signaling. In gastrocnemius muscle (Figure 1D), insulin increased Akt phosphorylation in the F0-C group, whereas this response was completely blunted in the F0-BPA10 group, consistent with severe insulin resistance. These results demonstrate that the lowest dose of BPA had considerable effects on glucose homeostasis and enhanced insulin resistance in both liver and muscle from pregnant mice. Consistent with this, we detected hyperinsulinemia in both F0-BPA10 and F0-BPA100 mice relative to F0-C mice (Table 1). F0-BPA100 mice also exhibited higher levels of plasma triglycerides and glycerol and increased plasma leptin compared with F0-C mice (Table 1).

### BPA exposure during pregnancy: consequences for mothers later in life

To determine whether BPA exposure during pregnancy is detrimental to glucose metabolism in females after parturition, we treated pregnant mice as described above and monitored metabolic parameters in these animals for several months postpartum. Three months after delivery, BPA-treated animals showed an increase of body weight that was more pronounced in the F0-BPA100 group [see Supplemental Material, Figure 1A (doi:10.1289/ehp.1001993)]. At this point of evaluation, we observed no statistically significant differences in insulin sensitivity in F0-BPA100 mice (see Supplemental Material, Figure 1B).

**Figure 2:**
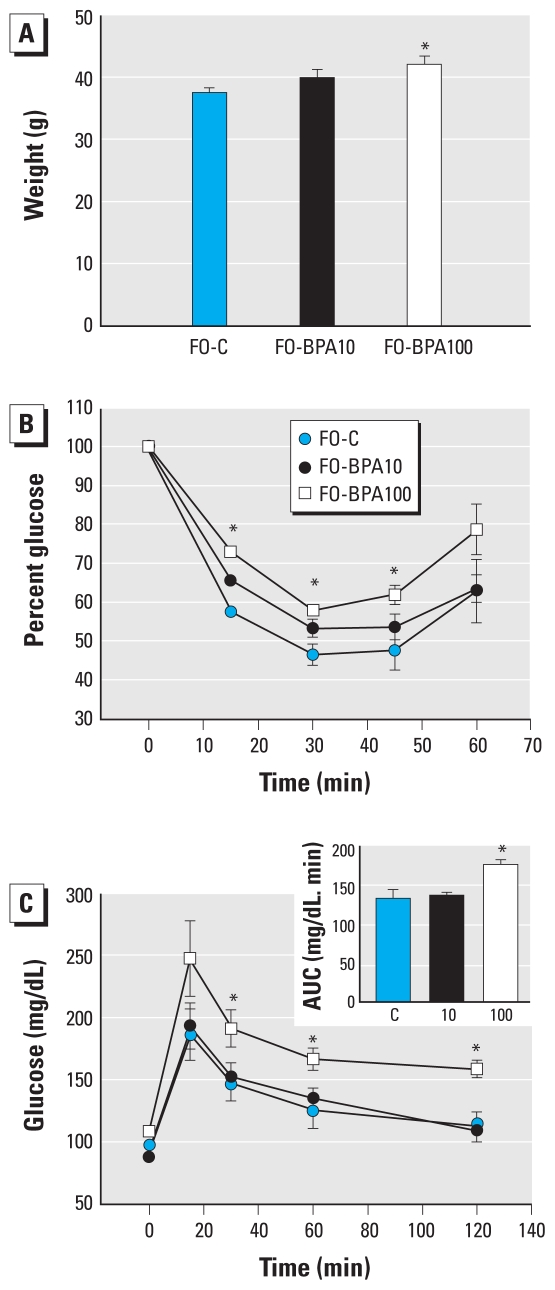
Analysis of glucose and insulin sensitivity in F0-C, F0-BPA10, and F0-BPA100 female mice (F0) 4 months after delivery. (*A*) Mean body weight. (*B*) IPITT. (*C*) IPGTT in the same mothers as in *B*; the inset represents the AUC. Data are expressed as mean ± SE (*n* = 6/group). *p < 0.05 for F0-BPA100 mice compared with F0-C.

Four months after delivery, the increased body weight associated with BPA exposure persisted in the F_0_ mice (Figure 2A); no differences in food intake were measured during a 6-day evaluation [see Supplemental Material, Figure 1C (doi:10.1289/ehp.1001993)]. When we performed the IPITT, the glucose-lowering effects of insulin were attenuated in F0-BPA100 compared with F0-C mice (Figure 2B). Consistent with this, we observed that glucose tolerance was clearly altered in F0-BPA100 mice (Figure 2C). Accordingly, in mice fasted 4 hr, plasma insulin levels were 2.2 times higher in the F0-BPA100 mice than in controls (Table 2), whereas glucose levels did not differ. These observations reinforced the idea that F0-BPA100 animals develop insulin resistance. Moreover, the higher plasma insulin levels were accompanied by increased plasma leptin, triglyceride, and glycerol levels relative to controls (Table 2). However, in the case of F0-BPA10, we observed no differences in IPITT and IPGTT results (Figure 2B,C), and only triglyceride levels were statistically different from those in control animals (Table 2). Nevertheless, plasma insulin, glycerol, and leptin levels were also increased, although the increases were not statistically significant (Table 2). Collectively, these results suggest that exposure to a BPA dose of 100 μg/kg/day during pregnancy has persistent metabolic consequences that, with time, lead to insulin resistance and dysregulated nutrient metabolism.

### BPA exposure during pregnancy: fetal programming of type 2 diabetes in adult offspring

To test whether BPA exposure *in utero* predisposes for future development of metabolic abnormalities, we studied three groups of animals: offspring from F0-C mothers (F1-C), offspring from F0-BPA10 mothers (F1-BPA10), and offspring from F0-BPA100 mothers (F1-BPA100). Note that these offspring received no direct treatment with BPA, but their mothers were treated GD9–GD16.

**Figure 3:**
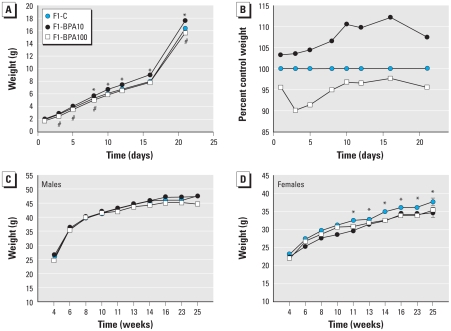
Body weight of F1-C, F1-BPA10, and F1-BPA100 mice. Mean body weight (*A*) and percentage of control weight (*B*) from birth to weaning for males and females combined. (*C, D*) Mean body weight from weaning to adulthood (22 through 180 days of age) for males (*C*) and females (*D*). *n* = 25–60 animals/group; some SEs are not visible because of their low values. *p < 0.05 for F1-BPA10 compared with F1-C. ^#^*p* < 0.05 for F1-BPA100 compared with F1-C.

F1-BPA10 mice were 3% heavier than F1-C mice at birth and 7% heavier at weaning (21 days postpartum). In contrast, body weight in the F1-BPA100 group was 4.5% lower than that of F1-C controls at birth, and the relative difference persisted until weaning (Figure 3A,B). In male offspring, body weight was comparable among experimental groups from weaning through 6 months of age (Figure 3C). In female offspring body weight was also comparable among groups in the postnatal period, but at 3 months of age F1-BPA10 and F1-BPA100 mice weighed 3% and 2.5% less, respectively, than did F1-C controls (Figure 3D).

When mice were 3 months of age, we performed IPITT to evaluate insulin sensitivity of the offspring. Insulin sensitivity was slightly decreased in F1-BPA10 male offspring compared with controls but not in F1-BPA100 offspring. Nevertheless, differences were not statistically significant [see Supplemental Material, Figure 2A (doi:10.1289/ehp.1001993)]. No differences were detected in female offspring (see Supplemental Material, Figure 2B).

**Figure 4:**
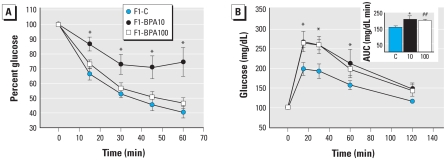
Blood glucose homeostasis in F1-C, F1-BPA10, and F1-BPA100 mice at 6 months of age shown by (*A*) IPITT and (*B*) IPGTT performed in the same group of animals. The inset in *B* represents the mean total glucose AUC. Data are expressed as mean ± SE (*n* = 8). *p < 0.05 for F1-BPA10 and F1-BPA100 mice compared with F1-C. ^##^*p* = 0.05.

In 6-month-old male mice, insulin sensitivity was clearly impaired in F1-BPA10 animals, as shown in Figure 4A, yet insulin sensitivity was normal in F1-BPA100 males. Glucose tolerance was altered in both F1-BPA10 and F1-BPA100 males: The AUC was significantly increased in F1-BPA10 and nonsignificantly increased in F1-BPA100 mice relative to F1-C controls (Figure 4B, inset). In 6-month-old female offspring, we found no differences in insulin sensitivity or glucose tolerance [see Supplemental Material, Figure 2C,D (doi:10.1289/ehp.1001993)]. Therefore, we performed subsequent studies only with male animals.

**Figure 5:**
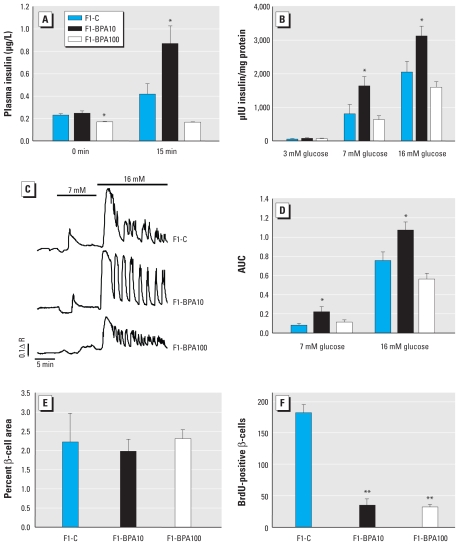
Pancreatic islet function in male F1-C, F1-BPA10, and F1-BPA100 mice. *In vivo* plasma insulin levels 15 min after a glucose load (2 g/kg; *A*) and *ex vivo* glucose-induced insulin secretion from isolated islets (3, 7, and 16 mM glucose; *B*); *n* = 8–10 animals/group. (*C*) [Ca^2+^]_i_ response of a representative islet of Langerhans in the presence of 3, 7 and 16 mM glucose applied for 10, 10, and 15 min, respectively (*n* = 10/group). (*D*) AUC for traces in *C*. (*E*) Measurement of β-cell area (area occupied by insulin-positive cells expressed as a percentage of the total area). (*F*) Quantification of BrdU incorporation in insulin-positive cells. Data are expressed as mean ± SE. *p < 0.05 compared with F1-C. **p < 0.005.

Serum insulin and glycerol levels were higher in F1-BPA10 male mice compared with F1-C males, but leptin levels were comparable (Table 3). Interestingly, in F1-BPA100 mice, only glycerol levels were significantly increased relative to controls. To relate these observations with alterations in the endocrine pancreas, we studied glucose-stimulated insulin secretion 15 min after a glucose load *in vivo* and *ex vivo*. Glucose-stimulated insulin secretion was doubled in F1-BPA10 mice relative to F1-C males but was comparable in F1-BPA100 mice (Figure 5A). After *ex vivo* exposure to high glucose concentrations (16 mM), islets from F1-BPA10 mice secreted 1.53 times more insulin than did islets from control mice, whereas islets from F1-BPA100 mice secreted slightly less insulin than did controls, although the difference was not statistically significant (Figure 5B). Enhanced insulin secretion in F1-BPA10 mice was accompanied by a greater increase in global intracellular Ca^2+^ entry after glucose stimulation. However, in the F1-BPA100 group, the increase in global Ca^2+^ entry was slightly less than the response observed in controls (not statistically significant; Figure 5C,D). We next studied whether pancreatic β-cell mass was altered in BPA-treated male mice. Although we found no change in β-cell area among the groups (Figure 5E), BrdU incorporation into β cells, a marker of proliferation, was markedly reduced in both F1-BPA10 and F1-BPA100 males relative to F1-C mice (Figure 5F).

## Discussion

In the present study, we have demonstrated that exposure to low doses of BPA during critical periods of life has adverse effects on glucose homeostasis and insulin sensitivity. When pregnant mice were treated with BPA on GD9–GD16, they developed glucose intolerance and elevated levels of plasma insulin, triglycerides, glycerol, and leptin compared with control pregnant mice. Interestingly, this brief exposure to BPA had long-term metabolic consequences for both the mothers and their male offspring.

### BPA exposure in mothers: consequences for glucose homeostasis during pregnancy

Maternal adaptation to pregnancy is mediated mainly by placental hormones, such as prolactin, placental lactogens, and steroid hormones, including estrogens (Nadal et al. 2009b). Estrogen signaling is emerging as a key pathway in glucose and lipid metabolism and may play an important role in the insulin resistance that occurs during pregnancy (Gonzalez et al. 2003). In the pancreas, estrogen receptors (ERs) are important in the regulation of insulin biosynthesis and release, a process that appears to counteract the increased insulin resistance associated with pregnancy (Nadal et al. 2009b). BPA is a xenoestrogen, and its estrogenic effect alters pancreatic β-cell function and induces glucose intolerance and insulin resistance in male mice (Alonso-Magdalena et al. 2006). Moreover, its effect in β cells is direct and occurs via ERα (Alonso-Magdalena et al. 2008). In addition, BPA binding to extranuclear ERα alters prolactin secretion (Wozniak et al. 2005). Therefore, alterations of estrogen signaling by BPA treatment during pregnancy would be expected to produce deleterious effects on the adaptation of the endocrine pancreas, pituitary, and other peripheral tissues.

Results of the present study indicate that, compared with F0-C mice, F0-BPA10 mice had impaired glucose tolerance, slightly increased fasting plasma insulin levels (1.38 times higher than in controls), and reduced insulin sensitivity in skeletal muscle and liver (as evidenced by the inability of insulin to promote phosphorylation of Akt at Thr^308^ in these tissues) (Alessi et al. 1996). Thus, the reduction in glucose tolerance observed in F0-BPA10 mice compared with controls may reflect an inability to increase insulin levels enough to compensate for the observed increase in insulin resistance. In contrast, higher fasting plasma insulin levels in F0-BPA100 mice (double the mean concentration in controls) may have partially compensated for the relative increase in insulin resistance that we also observed in F0-BPA100 mice, and may explain why F0-BPA100 mice tolerated a glucose challenge better than did F0-BPA10 mice. In addition, fasting plasma leptin levels were 2.1 times higher in F0-BPA100 mice than in F0-C mice. A major function of leptin is regulation of glucose metabolism and insulin sensitivity in muscle, liver, and endocrine pancreas (Tudurí et al. 2009; Wang et al. 2010). Leptin levels are increased during pregnancy, and leptin receptors are present in maternal tissue, placenta, and fetal tissue (Sagawa et al. 2002). Therefore, increased levels of leptin may affect energy metabolism in mothers as well as fetal growth. It will be of interest to determine whether BPA directly regulates leptin release from adipocytes, as is the case of adiponectin secretion (Hugo et al. 2008), or whether the observed hyperleptinemia is a consequence of the altered metabolic state of these animals.

### BPA treatment during pregnancy affects mother’s glucose metabolism later in life

Experimental and observational data suggest that gestational diabetes may increase a mother’s risk for obesity and type 2 diabetes later in life (Boloker et al. 2002; Reece et al. 2009). F0-BPA10 mice weighed more (not significant) and had higher plasma triglyceride levels 4 months after delivery than controls. It is important to note that a BPA dose of 10 μg/kg/day is 5 times lower than the dose considered completely safe during a lifetime by the U.S. EPA (2010). The effects observed in F0-BPA100 mice, only twice the level considered safe during lifetime by U.S. EPA (50 μg/kg/day), were even more striking. With the higher dose, pregnant mice demonstrated increased insulin resistance, decreased glucose tolerance, and significantly higher fasting plasma insulin, leptin, triglyceride, and glycerol levels than F0-C mice. In humans, these metabolic alterations are associated with an increased risk of type 2 diabetes and cardiovascular disease (Kahn et al. 2006). Interestingly, increased insulin resistance in F0-BPA100 mice was not evident 3 months after delivery but was evident 4 months after delivery, which suggests that insulin resistance resolved after parturition but was triggered several months later by a yet unknown mechanism.

Effects of EDCs, including those of BPA, are usually classified as “activational” and “organizational.” “Activational” refers to effects resulting from adult exposure, and they are generally considered to be reversible. “Organizational” refers to effects resulting from perinatal exposure, and it is well accepted that these effects can persist even in the absence of subsequent reexposure (Richter et al. 2007). In the present study, we observed an effect of BPA in adults that appeared during gestational exposure, disappeared after labor, and reappeared later in life. Thus, these results challenge the long-standing assumption that effects of exposure to endocrine disruptors during adulthood are reversible after exposure ceases. The mechanistic basis of these alterations is not yet known, although it is plausible that epigenetic modifications of maternal tissues may influence metabolism later in life and, perhaps, during subsequent pregnancies.

### BPA treatment during pregnancy: implications for offspring metabolism

It is well known that conditions experienced *in utero* can have lifelong effects on health, the “fetal plasticity” theory (Barker 1998; Gilbert and Epel 2009). Experimental and epidemiological studies in rodents and humans suggest that high or low birth weight is a risk factor of type 2 diabetes later in life (Gilbert and Epel 2009). Mice with low birth weight from malnourished mothers present glucose intolerance and β-cell failure during adulthood (Jimenez-Chillaron et al. 2005). *In utero* exposure to EDCs, such as diethylstilbestrol (DES), provokes various diseases in offspring during adulthood (McLachlan et al. 2001). Although information related to EDC-induced metabolic syndrome is sparse, it is known that uterine exposure to DES in mice induces high birth weight and obesity in adult offspring (Newbold et al. 2008, 2009). Moreover, *in utero* exposure to low doses (25 μg/kg/day) of BPA in rats is associated with high birth weight (Rubin and Soto 2009).

Our results demonstrate that exposure during pregnancy to 10 μg/kg/day BPA was associated with higher birth weight. This may have reflected effects on the metabolic state of the mothers (F0-BPA10)—primarily reduced glucose tolerance and increased plasma insulin levels—which are factors that may influence fetal growth (Reece et al. 2009). Offspring (F1-BPA10) also demonstrated reduced glucose tolerance, increased insulin resistance, and higher levels of plasma insulin and glycerol (an indicator of high free fatty acids). In addition, β cells from F1-BPA10 mice were more sensitive to extracellular glucose, both *in vivo* and *ex vivo*. This enhanced secretion of insulin may represent a homeostatic mechanism to compensate for an increase in peripheral insulin resistance. However, the incorporation of BrdU, which we used to assess β-cell proliferation, was significantly reduced in both groups of BPA-exposed offspring compared with F1-C mice. This seeming contradiction between preserved β-cell mass and impaired BrdU incorporation might be explained by the effects of BPA on β-cell turnover. Although adult β cells expand during adulthood to match peripheral demands for insulin, recent studies have shown that in fact β cells from aged mice reside in a mostly quiescent state and display a very low rate of turnover (Teta et al. 2005, 2007). Thus, it is plausible that *in utero* exposure to BPA alters cell-cycle machinery without provoking apoptosis of β cells, and given that insulin secretion is enhanced, diabetes can be avoided in these animals until the increased demand for insulin reaches a point where expansion of β cells is also required to compensate for insulin resistance.

Notably, the metabolic phenotype of F1-BPA100 is quite different from that of the F1-BPA10 group. F1-BPA100 mice had lower birth weights than the F1-C group. Because F0-BPA100 mice presented milder glucose intolerance and higher levels of leptin than F0-BPA10 mice, this may influence birth weight and therefore glucose homeostasis of offspring. In addition, F1-BPA100 mice presented milder glucose intolerance than F1-BPA10 mice but normal insulin sensitivity and plasma insulin levels that were not statistically different from controls. When we analyzed *in vivo* pancreatic β-cell function, we found that the insulin response to an intraperitoneal injection of glucose was reduced in F1-BPA100 mice relative to controls. *Ex vivo* experiments with isolated islets indicate that glucose-induced Ca^2+^ signals and insulin release were only slightly diminished with respect to controls. Therefore, F1-BPA100 animals presented glucose intolerance likely due to liver insulin resistance rather than to alterations in the β-cell stimulus–secretion coupling.

Only male offspring had altered glucose tolerance and insulin resistance; age- and treatment-matched female offspring displayed normal metabolic parameters. Females are protected against insulin resistance more than are males, in part because the presence of estrogens—within the physiological range—protect against diabetes in mice (Liu and Mauvais-Jarvis 2010; Louet and Mauvais-Jarvis et al. 2004). Moreover, at 3 months of age, neither males nor females presented insulin resistance or glucose intolerance. In a study of glucose tolerance, Ryan et al. (2010) described similar results until 15 weeks of age, concluding that perinatal exposure to BPA (0.25 μg/kg/day) did not affect glucose homeostasis in adult mice. However, in the present study, although the BPA treatment protocol was different, metabolic alterations did not appear until later in adulthood (6 months of age).

The “developmental” or “fetal” origin of adult disease hypothesis states that environmental factors act early in life to program the risks of developing chronic diseases in adult life. In our model, metabolic effects observed in mice prenatally exposed to BPA may be due to two factors: abnormal hormonal environment and altered glucose metabolism. Whether the effects we observed in the offspring are due to a direct effect of BPA on the fetus or because the fetus is exposed to an altered maternal metabolism, or the combination of both factors, remains unknown. Nevertheless, the latter is the most plausible situation because BPA crosses the placenta and because glucose tolerance, insulin, and leptin signaling during gestation are important for fetal growth. Indeed, maternal metabolism is important because the different impaired glucose tolerance and blood parameters observed in F0-BPA10 and F0-BPA100 mice may explain, at least in part, the different metabolic abnormalities displayed subsequently in their offspring (F1-BPA10 and F1-BPA100 mice, respectively).

In any case, the results of the present study suggest that the endocrine disruptor BPA should be evaluated as a possible risk factor for gestational diabetes, type 2 diabetes, and cardiovascular disease associated with metabolic syndrome. Moreover, our findings in mice suggest that fetal exposure to BPA may predispose males to type 2 diabetes during adulthood.

## Correction

In the manuscript originally published online, the the units given in the tables for leptin levels (mg/mL) should have been ng/mL. they have been corrected here.

## Figures and Tables

**Table 1:** Plasma hormone and metabolite levels after 4 hr of fasting in pregnant mice on GD18 (*n* = 8–13 mice/group).

End point	F0-C	F0-BPA10	F0-BPA100
Insulin (μg/L)	0.82 ± 0.13	1.13 ± 0.15*	1.99 ± 0.37**
Triglycerides (mg/mL)	1.07 ± 0.15	1.71 ± 0.25	2.04 ± 0.36*
Glycerol (mg/mL)	0.33 ± 0.04	0.55 ± 0.07	0.87 ± 0.15*
Leptin (ng/mL)	1.2 ± 0.15	1.35 ± 0.11	2.52 ± 0.31*

* *p* < 0.05, and

** *p* < 0.005, compared with F0-C, by one-way ANOVA followed by Bonferroni.

**Table 2:** Plasma hormone and metabolite levels after 4 hr fasting in F0 mice 4 months after delivery (*n* = 6 mice/group).

End point	F0-C	F0-BPA10	F0-BPA100
Insulin (μg/L)	0.94 ± 0.20	1.61 ± 0.17	2.08 ± 0.39*
Triglycerides (mg/mL)	0.44 ± 0.07	1.22 ± 0.29#	1.97 ± 0.30#
Glycerol (mg/mL)	0.27 ± 0.03	0.66 ± 0.17	0.94 ± 0.19*
Leptin (ng/mL)	2.70 ± 0.59	5.33 ± 1.12	6.05 ± 1.06*

* *p* < 0.05, compared with F0-C, by one-way ANOVA followed by Bonferroni.

# *p* < 0.05, compared with F0-C, by one-way ANOVA followed by Holm-Sidak.

**Table 3:** Plasma hormone and metabolite levels after 4 hr of fasting in male offspring at 6 months of age (*n* = 6–13 mice/group).

End point	F1-C	F1-BPA10	F1-BPA100
Insulin (μg/L)	0.85 ± 0.13	1.73 ± 0.25*	1.40 ± 0.18
Triglycerides (mg/mL)	0.84 ± 0.16	1.41 ± 0.03	1.25 ± 0.09
Glycerol (mg/mL)	0.18 ± 0.02	0.27 ± 0.02#	0.24 ± 0.02#
Leptin (ng/mL)	1.69 ± 0.42	1.78 ± 0.47	1.85 ± 0.44

* *p* < 0.05, compared with F1-C mice, by one-way ANOVA followed by Bonferroni.

# *p* < 0.05, compared with F1-C mice, by one-way ANOVA followed by Holm-Sidak.
